# Erratum: A two-qubit photonic quantum processor and its application to solving systems of linear equations

**DOI:** 10.1038/srep42653

**Published:** 2017-05-12

**Authors:** Stefanie Barz, Ivan Kassal, Martin Ringbauer, Yannick Ole Lipp, Borivoje Dakić, Alán Aspuru-Guzik, Philip Walther

Scientific Reports 4: Article number: 611510.1038/srep06115; published online: 08 19 2014; updated: 05 12 2017

This article was originally published under a [CC BY-NC-SA 4.0/CC BY-NC-ND 4.0] license, but has now been made available under a [CC BY 4.0] license. The PDF and HTML versions of the paper have been modified accordingly.

